# Fluorine-doped graphene with an outstanding electrocatalytic performance for efficient oxygen reduction reaction in alkaline solution

**DOI:** 10.1098/rsos.180925

**Published:** 2018-10-03

**Authors:** Jiahao Guo, Jianguo Zhang, Hanqing Zhao, Yongshuang Fang, Kun Ming, Hao Huang, Junming Chen, Xuchun Wang

**Affiliations:** College of Chemistry and Materials Engineering, Anhui Science and Technology University, Fengyang, Anhui 233100, People's Republic of China

**Keywords:** fluorine-doped graphene, electrocatalytic performance, oxygen reduction reaction, alkaline solution

## Abstract

Doping carbon materials have proved to be the front runners to substitute for Pt as oxygen reduction reaction (ORR) catalysts. Fluorine-doped graphene (FG) has rarely been used as ORR catalyst because of the difficulty in preparation. Herein, we report FG sheets prepared by a thermal pyrolysis graphene oxide (GO) process in the presence of zinc fluoride (ZnF_2_) as an efficient electrocatalyst for ORR in the alkaline medium. The results show that the pyrolysis temperature seriously affected the doped fluoride amount and morphology of catalyst. It is found that the FG-1100 catalyst possesses a more positive onset potential, higher current density and better four-electron process for ORR than other FG samples. FG-1100 displays an outstanding ORR catalytic activity that is comparable to that of the commercial Pt/C catalyst. Also, its durability and methanol tolerance ability are superior to those of the commercial Pt/C. The excellent ORR catalytic performance is closely related to its higher doped fluorine amount and wrinkle morphology. The FG catalyst can be developed as a low-cost, efficient and durable catalyst as a viable replacement for the Pt/C catalyst, promoting the commercialization of fuel cells.

## 1. Introduction

Oxygen reduction reaction (ORR) plays a pivotal role in clean energy conversion applications such as fuel cells and metal-air batteries [1–3]. The sluggish kinetics of ORR is a major limitation in the efficiency and performance of such devices, compromising their widespread use [4–7]. This issue motivates the search for electrocatalysts with improved ORR performance [8–12]. Although platinum (Pt) exhibits the best performance for ORR to date, the high cost, scarcity and susceptibility to agglomeration and deactivation are gargantuan obstacles to the large-scale commercial production for energy devices [13]. Therefore, exploring economic, stable and effective non-precious metal [14,15] or metal-free substitutes [16–21] for Pt ORR catalyst has become a topic of interest.

In recent years, carbon-based materials have proved to be front runners to substitute for Pt as ORR catalysts, possessing excellent catalytic activity and good resistance to methanol crossover effects [22]. Theoretical calculation has shown that introducing heteroatoms into the sp^2^-hybridized framework of carbon to regulate the surface electronic structure plays a key role in the improvement of catalytic performance for ORR [23–25]. The difference of electronegativity (*χ*) between heteroatom and carbon atom in carbon frameworks leads to charge imbalance and polarizes adjacent carbon atoms, creating net positive/negative charges, which is favourable for ORR. Thus, carbon-based materials doped with heteroatoms, such as boron (B, 2.04), [26,27] phosphorus (P, 2.19), [28,29] nitrogen (N, 3.04), [30–35] sulfur (S, 2.58) [36–39] and selenium (Se, 2.55) [40] have attracted tremendous attention. As elements with larger electronegativity, halogens, including chlorine (Cl, 3.16), bromine (Br, 2.96) and iodine (I, 2.66), are also applied to ORR catalysts as heteroatoms for carbon materials. Yao *et al*. [41] prepared iodine-doped graphene (IG) through heat treatment of graphene oxide (GO) and I_2_ mixtures; the IG exhibited high catalytic activity, good stability and strong resistance to methanol crossover ability in alkaline electrolyte. In IG, iodine exists in the form of I_3_^−^ and I_5_^−^, in which I_3_^−^ plays an important role in improving the ORR activity. Jeon *et al*. [42] synthesized a series of halogenated graphene nanoplatelets (ClGnP, BrGnP and IGnP) by simply ball-milling graphite flake in the presence of chlorine (Cl_2_), bromine (Br_2_) or iodine (I_2_), respectively. XGnPs showed remarkable electrocatalytic activities toward ORR with a high selectivity, good tolerance to methanol crossover/CO poisoning effects and excellent long-term cycle stability. They thought the edge-halogenations play an important role in significantly improving the ORR activity of graphite. As fluorine (F_2_) is too reactive to be handled in normal laboratories and fluorine ion (F^−^) is very stable, it is difficult to prepare fluorine-doped carbon-based materials. Few research works have been done to apply fluorine-doped carbon-based materials as ORR catalyst.

Herein, we have developed a facile synthesis of metal-free fluorine-doped graphene sheets (FG) as an efficient ORR electrocatalyst simply by a thermal pyrolysis GO process in the presence of zinc fluoride (ZnF_2_). It is found that the resultant FG catalyst exhibits outstanding catalytic activity for ORR, which is comparable to that of the commercial Pt/C catalysts at the same condition, together with excellent stability and high methanol tolerance. The results not only address a new, low-cost, mass production FG graphene as metal-free efficient ORR catalyst for fuel cells but also provide useful information to enucleate ORR mechanisms of carbon-based materials doped with heteroatoms.

## 2. Experimental

### 2.1. Preparation of the F-doped graphene sheets

GO was purchased from the Sixth Element Material Ltd (Changzhou) without further treatment. F-doped graphene sheets were prepared by directly annealing graphene sheets and ZnF_2_ in argon. In a typical procedure, GO (30 mg) and zinc fluoride (300 mg) were firstly dispersed in deionized water (100 ml) by ultrasonic irradiation for 6 h. After resting for 12 h, the suspension was washed and centrifuged with deionized water, then transferred to an evaporating dish and dried at 50°C under vacuum. The obtained powder mixture was pyrolyzed at 1000°C, 1100°C and 1200°C in Ar atmosphere for 2 h to obtain FG-1000, FG-1100 and FG-1200, respectively. For comparison, GO was pyrolysed at 1100°C in Ar for 2 h to produce a control sample, denoted as G-1100.

### 2.2. Material characterization

X-ray powder diffraction (XRD) profiles were obtained on a Rigaku D/max-rA with Cu K*α* radiation (*λ* = 1.54178 Å) at a scan rate of 8° min^−1^ with a step of 0.02°. Raman spectra were recorded on a Senterra R200-L Raman microscope, using a diode laser with excitation at 532 nm. Calculation of the parameters *I*_D_/*I*_G_ (integrated intensity ratio) was done by the deconvolution of the spectra. The morphology of the samples was examined by the images of scanning electron microscopy (SEM) and transmission electron microscopy (TEM). SEM images were acquired from an FEI HITACHI S-4800 field emission scanning electron microscope. TEM images were taken with a JEOJ-2010 TEM with an acceleration voltage of 200 kV. X-ray photoelectron spectra (XPS) were performed with an ESCALab MKII using Mg K*α* (1253.6 eV) radiation exciting source. Binding energies for the high-resolution spectra were calibrated by setting C1s to 284.4 eV.

### 2.3. Preparation of the working electrode

Typically, 4 mg FG powder was added into 1 ml mixture of deionized water and isopropanol with 17 µl Nafion (5 wt%, DuPont). The volume ratio of deionized water and isopropanol was 2.5 : 1. The solution was ultrasonicated for 1 h to form a homogeneous ink. Subsequently, 5 µl of the catalyst ink was introduced onto the clear glass carbon electrode (GC) of rotating disc electrode (RDE) or rotating ring-disc electrode (RRDE) (5 mm in diameter, Pine Research Instrument) by using a drop casting method. The GC with catalyst ink was dried at room temperature. For comparison, a commercial Pt/C catalyst (20 wt% Pt on carbon black, Johnson Matthew) ink was prepared by the same procedure described above. The loading of various catalysts was approximately 0.1 mg cm^−2^.

### 2.4. Electrochemical tests

The ORR performances of FG catalyst were evaluated by electrochemical methods, including cyclic voltammograms (CV), RDE voltammograms, RRDE voltammograms and chronoamperometry, using a CHI 660E electrochemical workstation (Chenhua, Shanghai) in a conventional three-electrode system at room temperature. An RDE coated with the catalyst film was used as the working electrode, a Pt wire as counter electrode and an Ag/AgCl (PINE, 4 M KCl) as reference electrode. 0.1 M KOH solution was employed as the electrolyte. The measured potentials were converted to the reversible hydrogen electrode (RHE) according to the Nernst equation:

$$E_{\mathrm{RHE}}=E_{\mathrm{Ag}/\mathrm{AgCl}}+0.21\;V+0.0592\;\mathrm{pH},$$

where *E*_Ag/AgCl_ is the experimentally measured potential versus Ag/AgCl. The potential provided in the text was referenced against RHE. The electrolyte was saturated with oxygen by bubbling O_2_ prior to the start of each experiment. The O_2_/Ar was continuously injected into the cell to maintain saturation during the electrochemical measurements. CV was performed from 0 to 1.2 V versus RHE with a scan rate of 10 mV s^−1^. RDE measurement was scanned cathodically from 1.2 to 0 V versus RHE at a rate of 10 mV s^−1^ under disc rotation rates of 400, 625, 900, 1225, 1600, 2025 and 2500 r.p.m. The chronoamperometry was carried out at 0.7 V at a rotation speed of 1600 r.p.m.

The electron transfer number (*n*) during ORR was determined by the Koutecky–Levich (K–L) equation [43]:

2.1 $$\frac{1}{J}=\frac{1}{J_{K}}+\frac{1}{B\varpi^{1/2}}$$

and

2.2 $$\;B=0.2nF{\left(D_{O}\right)}^{2/3}\nu^{-1/6}c_{O},$$

where *J* is the measured current, *J*_K_ is the kinetic current, *ω* is the electrode rotating rate, *n* represents the electron transfer number per oxygen molecule. *F* is the Faraday constant (96 485 C mol^−1^). *D_O_* is the diffusion coefficient of O_2_ in 0.1 M KOH (1.9 × 10^−5^ cm^2^ s^−1^). *ν* is the kinetic viscosity (0.01 cm^2^ s^−1^). *C*_O_ is the bulk concentration of O_2_ (1.2 × 10^−6^ mol cm^−3^). The constant 0.2 is adopted when the rotation speed is expressed in r.p.m.

In RRDE tests, catalyst inks and electrodes were prepared by the same method as RDEs. The disc electrode was scanned cathodically at a rate of 10 mV s^−1^ with 1600 r.p.m. and the Pt ring potential was constant at 1.25 V versus RHE to oxidize HO_2_^−^ intermediate from the disc electrode. The HO_2_^−^% and the electron transfer number (*n*) were determined by the following equations [44,45]:

2.3 $${\mathrm{HO}}_{2}^{-}\text{\%}=\frac{200\times I_{r}}{NI_{d}+I_{r}}$$

and

2.4 $$\;n=\frac{4\times I_{d}}{I_{d}+I_{r}/N},$$

where *I*_d_ is the disc current, *I*_r_ is the ring current and *N* is the current collection efficiency of the Pt ring and is 0.39 in our experiment.

## 3. Results and discussion

### 3.1. Material synthesis and characterization

**Figure 1.**
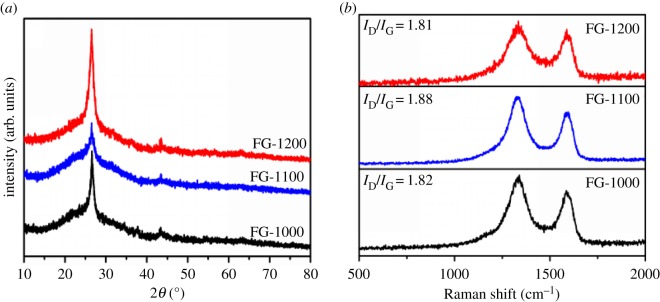
(*a*) XRD spectra and (*b*) Raman spectra of FG samples.

To investigate the crystal structure of FG, XRD was carried out and the results are shown in figure 1*a*. Two peaks are found in the XRD pattern of the FG-1000 sample which are located at 26.5° and 43.4°, corresponding with the (002) and (100) plane of graphite 2H (PDF #75-1621). As the pyrolysis temperature rose to 1100°C, the diffraction peaks of FG-1100 became weaker, suggesting less degree of graphitization. After further increasing the temperature to 1200°C, the two diffraction peaks became sharper again, meaning that the high temperature pyrolysis showed an improved graphitization degree. The (002) diffraction peak had no obvious shift or broadening after doping with fluorine, indicating no change in the interlayer spacing of graphene after doping [20]. This phenomenon can be explained by the low doping content of fluorine into the graphite interlayer, which was consistent with the XPS result. The crystallite sizes of FG-1000, FG-1100 and FG1200 calculated by the Scherrer equation based on the (002) peak are 54, 36 and 61 nm, respectively.

Raman spectroscopy is an effective means to characterize the structural information of carbon materials and it was used in this study to determine the defect sites in the range of Raman shift from 500 to 3200 cm^−1^ as shown in figure 1*b*. The Raman spectra of the FG samples all exhibited two prominent signals with their peaks located at around 1339 cm^−1^ and 1587 cm^−1^, corresponding to the D band and the G band, respectively. The D band is derived from lattice distortions in the hexagonal sp^2^-carbon network, which indicates the presence of disordered carbon atoms [46]. The G band is associated with the structural intensity of the sp^2^-hybridized carbon atom. The ratio of the intensity of D/G bands is used to measure the density of defects in the graphene layer [47,48]. As seen in figure 1*b*, the integrated *I*_D_/*I*_G_ ratio values of FG-1000, FG-1100 and FG-1200 are 1.82, 1.88 and 1.81, respectively. The *I*_D_/*I*_G_ value of FG-1100 is greater than that of the other two samples, which means a higher structural defectiveness. This is caused by the destruction of the graphene framework by the fluorine dopant and also shows that the fluorine doping amount of FG-1100 is the highest, which has been proved by the XRD result. But the *I*_D_/*I*_G_ ratio value of three FG samples is close to that of graphene, [49] which indicates that the F-doping amount in all FG samples is lower and the destruction of graphene framework is smaller. This result is confirmed by the later XPS characterization.

**Figure 2.**
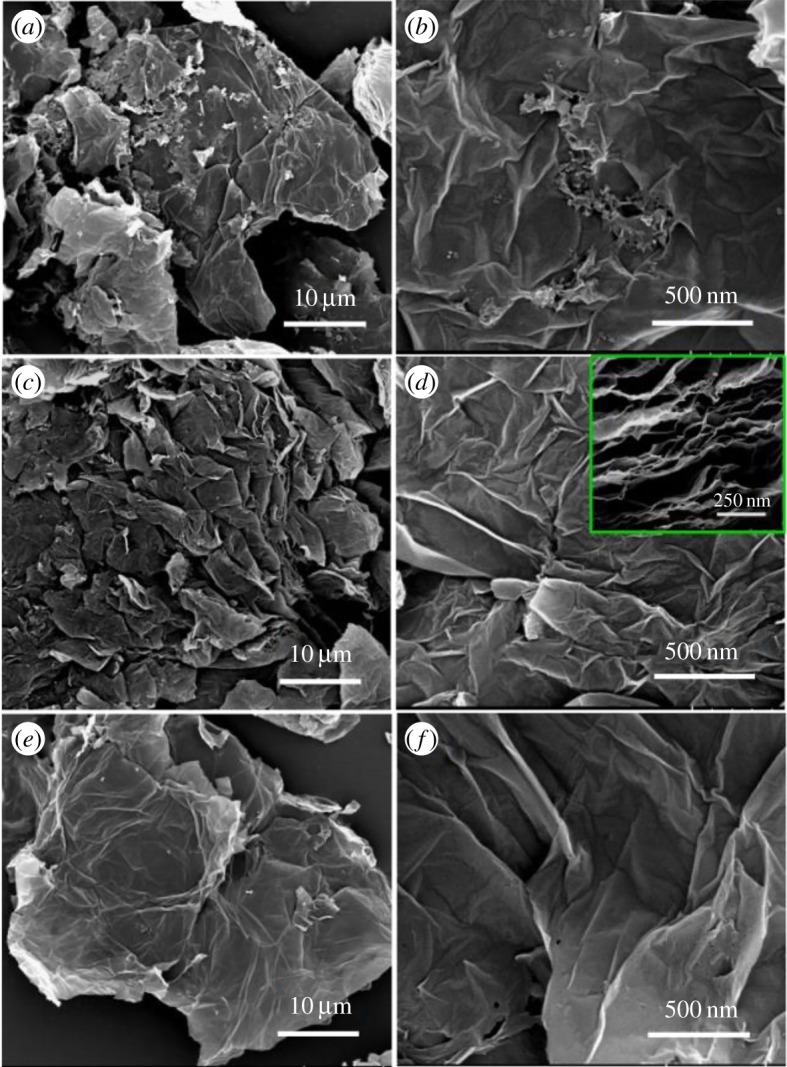
SEM images of FG-1000 (*a,b*), FG-1100 (*c,d*) and FG-1200 (*e,f*).

**Figure 3.**
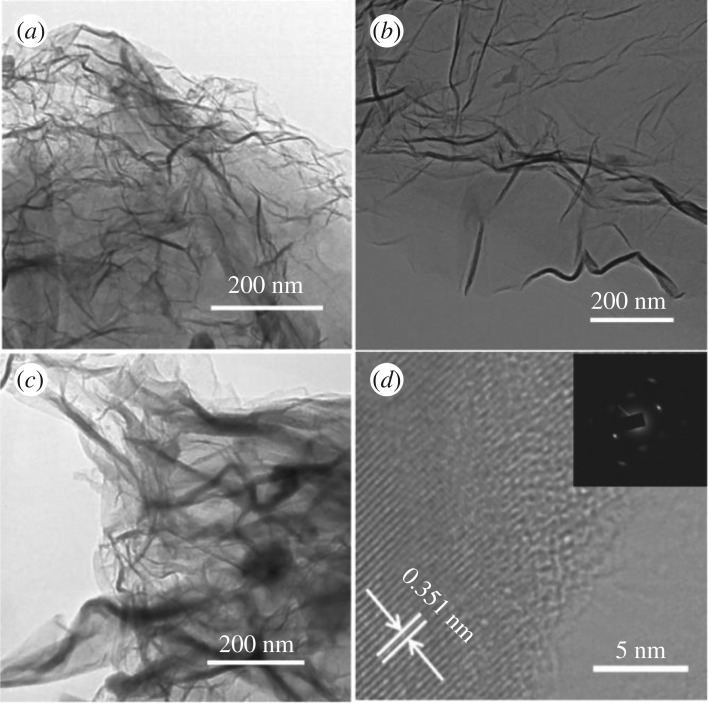
TEM images of FG-1000 (*a*), FG-1100 (*b*), FG-1200 (*c*) and HRTEM of FG-1100 (*d*). The inset in (*d*) is the corresponding selected area electron diffraction (SAED) pattern.

The morphology and microstructure of the FG samples were characterized by SEM and TEM. The SEM images as shown in figure 2 present highly wrinkled sheet-like morphology of the FG samples. There are many irregular nanoparticles on the surface of FG-1000. When the pyrolysis temperature was increased to 1100°C, the surface of graphene became very clean and the crumpled structure became more obvious, leading to a large number of corners and edges on the sample surface. This structure is beneficial to the doping of fluorine and the adsorption of reactant particles, which is conducive to the improvement of catalytic activity. As can be seen from the inset image in figure 2*d*, the FG-1100 sample is made up of the interlaced ultrathin nanosheets. Such a structural feature can provide large specific area, several exposed active sites and convenient fluid penetration, which are highly desirable for electrocatalysis. This conjecture was confirmed by the result of nitrogen adsorption–desorption experiment. The Brunauer–Emmett–Teller (BET) surface area of FG-1100 is 182 m^2^ g^−1^, a relatively large surface area, and the pore size is mainly centred at approximately 23 nm (electronic supplementary material, figure S2). For the FG-1200 sample, the surface becomes flat and the wrinkles decrease. The TEM results validate this conclusion. The TEM images of all FG samples present the ultrathin, transparent and wrinkled shape of typical graphene morphology. The high-resolution transmission electron microscopy (HRTEM) image of FG-1100 in figure 3*d* disclosed a few-layer structure of the FG sheets and displayed an inter-planar spacing of 0.351 nm, corresponding to the (001) plane of graphene. The electron diffraction pattern (the inset of figure 3*d*) exhibits diffraction points rather than diffraction circles, testifying an exfoliated thin layer of FG-1100 by high-temperature treatment.

**Figure 4.**
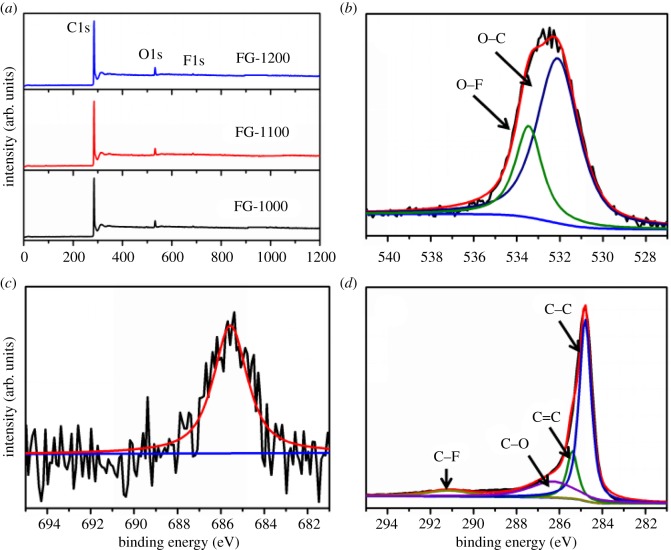
(*a*) XPS spectra of the FG samples, (*b*) XPS-O1s, (*c*) XPS-F1s and (*d*) XPS-C1s spectra of FG-1100.

X-ray photoelectron spectroscopy (XPS) was carried out to analyse the chemical state and composition of the elements. As shown in figure 4*a*, the XPS survey spectra of FG samples showed C1s, O1s and F1s signals at approximately 284, 532 and 685 eV, respectively, indicating the presence of carbon, oxygen and fluorine elements in the FG samples. The appearance of F1s peak proves that fluorine has been successfully incorporated into the skeleton of graphene after pyrolysis of the GO and ZnF_2_ mixture. During heating, Zn^2+^ was reduced to the metallic state by carbon firstly, then Zn was evaporated at the pyrolysis temperature, thus Zn can be removed thoroughly. The indicated C, O and F contents of the FG samples are shown in table 1. It is found that the total fluorine content of FG-1100 is 2.61 at.%, higher than that of FG-1000 (1.88 at.%) and FG-1200 (2.45 at.%). In the deconvolved high-resolution O1s spectrum for FG-1100 (figure 4*b*), two chemical bonding states of O can be indexed, O–C (531.9 eV) and O–F (533.6 eV). The deconvolved F1s spectrum in figure 4*c* shows one peak, located at 685.6 eV, which can be attributed to the F–C bond [50]. Owing to the low F content, great noise exists in the high-resolution F1s peak, especially for FG-1000 (electronic supplementary material, figure S1). In figure 4*d* and electronic supplementary material, figure S1, the C1s spectrum can be resolved into four peaks at the binding energies of 284.8, 285.2, 286.3 and 291.2 eV, corresponding to the sp^2^-carbon (C–C), sp^3^-carbon (C=C), hydroxyl (C–O) and C–F bonding configurations, respectively. The relative atomic contents of the surface functional groups obtained from the deconvolution of C1s are summarized in table 1. The total contents of sp^2^-carbon (C–C) and sp^3^-carbon (C=C) are close to 75 at.%, indicating that GO was effectively reduced through pyrolysis treatment. Although the fluorine content is the highest, the content of C–F bond for FG-1100 is lower than those of the other samples and only reaches 7.07%. This is because there are many edges on the surface of FG-1100. The doping F element is mainly located at the edges. However, FG-1100 possesses a porous structure arising from the interlaced ultrathin nanosheets. F element can penetrate into the pores in the doping process and cannot be detected, leading to the decrease of the surface content. These edge-doped F are catalytical active sites that can efficiently adsorb and catalyse ORR, thus enabling FG-1100 catalysts to have excellent ORR catalytic properties.

**Table 1.** The normalized atomic percentage of O, F, C and different C configurations in each FG sample.

sample	O (at.%)	F (at.%)	C (at.%)	C–C (at.%)	C=C (at.%)	C–O (at.%)	C–F (at.%)
FG-1000	5.14	1.88	92.98	46.16	23.32	12.90	10.60
FG-1100	5.01	2.61	92.38	52.97	13.44	18.90	7.07
FG-1200	4.96	2.45	92.59	57.91	14.37	11.80	8.51

### 3.2. Electrocatalytic analysis

**Figure 5.**
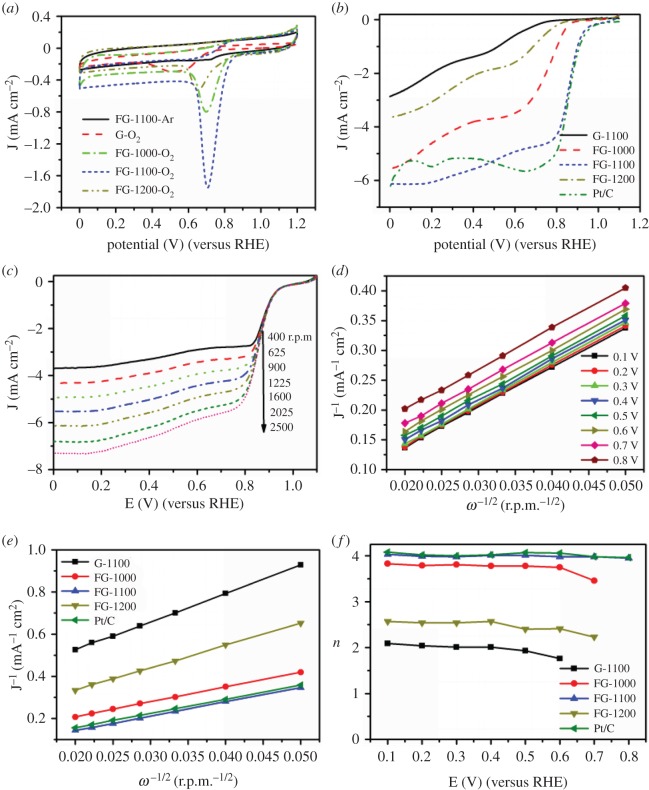
(*a*) CVs of FG samples and Pt/C in Ar- and O_2_-saturated 0.1 M KOH solution with 10 mV s^−1^. (*b*) LSV of FG samples and Pt/C in O_2_-saturated 0.1 M KOH with an RDE rotation rate of 1600 r.p.m. and 10 mV s^−1^. (*c*) LSV of FG-1100 at different RDE rotation rates. (*d*) Calculated K–L plots of ORR from FG-1100. (*e*) K–L plots of ORR from FG samples and Pt/C at 0.3 V. (*f*) Electron transfer number derived from K–L plots at different potentials.

The ORR activity of the FG samples was first evaluated in a three-electrode cell configuration through cyclic voltammetry (CV) in Ar- and O_2_-saturated 0.1 M KOH solution at room temperature and was compared against the performance of a commercial Pt/C catalyst. CV curves obtained in the Ar- and O_2_-saturated 0.1 M KOH on different electrodes are shown in figure 5*a*. Featureless voltammetric curve can be observed in Ar-saturated solution, indicating that there are no active redox species presenting in the electrolyte in the potential window from 0 to 1.2 V versus RHE. When the electrolyte was saturated with O_2_, a well-defined cathodic peak can be observed for all F-doping graphene and G-1100, strongly indicating that the G-1100 and FGs can act as a catalyst for ORR in the alkaline medium. G-1100 sample shows a negligible current density compared to other catalysts and a slight peak potential of 0.514 V (versus RHE) demonstrates low ORR activity. The oxygen reduction peaks progressively shifted to more positive potentials and the oxygen reduction current increased for FG samples. Significant increase in the peak current density was found after the pyrolysis of the mixture of GO and ZnF_2_ in Ar, and distinct ORR peaks appeared for FG-1000, FG-1100 and FG-1200 at the potential of about 0.695 V, 0.725 V and 0.654 V, respectively. The improvement of ORR activity is attributed to efficient fluorine doping in high temperature under Ar condition. The most positive ORR potential is observed for FG-1100 at 0.725 V along with a peak current density of 1.67 mA cm^−2^ (figure 5*a*), after correcting background current density, which compared favourably with 0.825 V and 1.05 mA cm^−2^ determined for the Pt/C reference electrocatalyst (electronic supplementary material, figure S3). The results confirmed that FG-1100 sample possessed excellent electrocatalytic activity for ORR. Based on the above results, both the fluorine doping and the control of temperature are important factors for ORR activity.

A detailed investigation of the electrocatalytic performance of the FG samples was carried out by experiments using an RDE and an RRDE. The linear scan voltammogram (LSV) curves obtained from RDE with 1600 r.p.m. and 10 mV s^−1^ in O_2_-saturated 0.1 M KOH are shown in figure 5*b*. To provide a realistic picture, the results are compared with those of a commercial Pt/C catalyst with the same amount of each catalyst. As shown in figure 5*b*, the limiting current densities for the G-1100, FG-1000, FG-1100 and FG-1200 samples at 0.2 V versus RHE were 2.01, 4.63, 6.07 and 3.07 mA cm^−2^, respectively. The onset potentials for ORR were 0.792, 0.911, 0.991 and 0.875 V, respectively, shifting progressively toward more positive potentials from G-1100 to FG-1100. This same trend was also seen in the half-potentials (0.596, 0.742, 0.860 and 0.688 V, respectively). Obviously, FG-1100 sample displayed the best ORR performance, with a similar half-potential (0.860 V), onset potential (0.991 V) and superior limiting current density (6.07 mA cm^−2^) compared to the commercial Pt/C catalyst (0.856, 1.005 and 5.49 mA cm^−2^, respectively). It should be noted that the catalytic performance of FG-1100 catalysts is close to or even superior to those of doped carbon materials with outstanding performance for ORR reported in the recent literature in terms of onset potential, half-wave potential, current density as well as *n* as shown in electronic supplementary material, table S1. The better catalytic performance of FG-1100 can be attributed to the intrinsic properties originated from the F-doping effect, together with exposed edges and the nature of the crumpled structure of the layer sheet, which have been confirmed by Raman spectra, SEM image and XPS analyses in figures 1, 2 and 4, respectively. The Raman spectra indicated the presence of more defects in FG-1100 than in other samples and can provide more exposed edges for oxygen adsorption and more ORR active sites. The interlaced ultrathin nanosheet structure that emerged from the SEM image is convenient for the transfers of reactant to and from the electrolyte. XPS results showed the high amount of fluorine in FG-1100, which plays an important role in improving ORR. The Tafel slope of FG-1100 at low over-potential is much smaller than those of G-1100, FG-1000 and FG-1200, close to that of Pt/C catalyst, meaning excellent ORR activity (electronic supplementary material, figure S4). We also optimized the doping amount of FG-1100 by adding a different amount of ZnF_2_ and investigated the influence of doping amount for catalytic activity of FG-1100. It can be seen from electronic supplementary material, figure S5 that FG-1100 catalyst doped with 300 mg ZnF_2_ occupied an optimal catalytic activity.

To further explore the ORR kinetics, the K–L analysis was performed by rotating disc electrode (RDE) measurement at rotating rates from 400 to 2500 r.p.m. (figure 5*c*; electronic supplementary material, figure S6). K–L plots were calculated from LSV curves at different potentials and all plots for FG-1100 showed good linearity, indicating the first-order reaction kinetics toward the concentration of O_2_ (figure 5*d*; electronic supplementary material, figure S6). The electron transfer number (*n*) for ORR on FG-1100, derived from the slopes of K–L plots, which were 3.95–4.01, exhibits dominant four-electron process for ORR. Similar results of the commercial Pt/C catalyst were found (electronic supplementary material, figure S6). The LSV curves and K–L curves for G-1100, FG-1000 and FG-1200 are presented in electronic supplementary material, figure S6, and produce *n* values of 1.76–2.04, 3.46–3.81 and 2.23–2.57, respectively, indicating a reduction process with combined two-electron and four-electron reaction pathways for ORR. FG-1100 presented a higher kinetic current density (33–52 mA cm^−2^) determined from the intercept of K–L plots than those of G-1100, FG-1000 and FG-1200 (3–8, 19–32, 12–25 mA cm^−2^, respectively). These results confirmed the excellent ORR catalytic performance of FG-1100.

**Figure 6.**
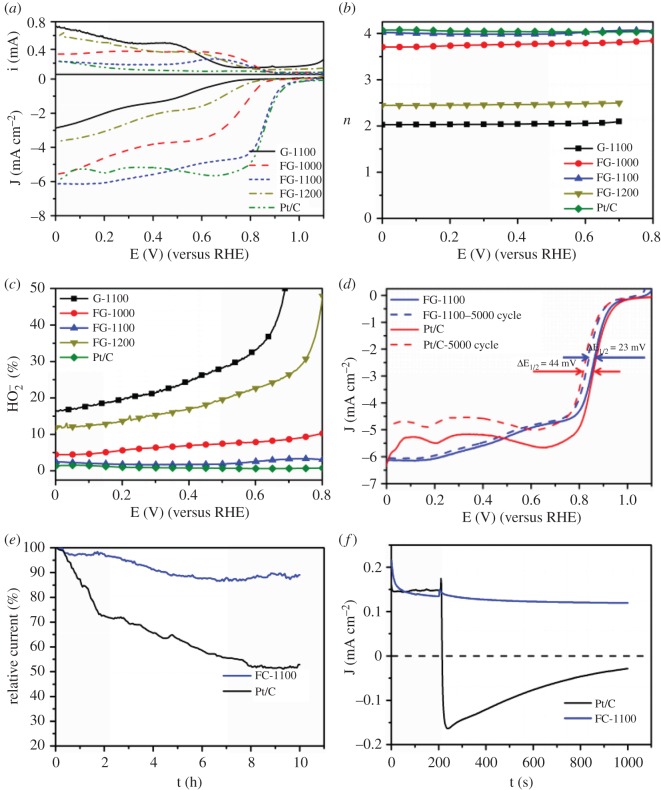
(*a*) RRDE measurement of FG samples and commercial Pt/C catalysts for ORR. (*b*) The number of electrons transferred per O_2_ as a function of potential for the catalysts. (*c*) Calculated HO_2_^−^ production yields of the catalysts during the ORR. (*d*) LSV curves of FG-1100 and Pt/C before and after 5000 cycles. (*e*) Chronoamperometric response of FG-1100 and Pt/C. (*f*) *i*–*t* of FG-1100 and Pt/C before and after the addition of 3 M methanol. Tests were conducted in O_2_-saturated 0.1 M KOH solution at 0.6 V.

To verify the ORR catalytic pathway, a more in-depth research was carried out with RRDE measurements to accurately monitor the formation of peroxide species (HO_2_^−^) and the 4e^−^ selectivity of the FG samples during the ORR process. Figure 6*a* displays the representative ORR polarization curves of all FG samples with 1600 r.p.m. in O_2_-saturated 0.1 M KOH. The catalytic activity for ORR increased in the sequence of G-1100, FG-1200, FG-1000, FG-1100 and Pt/C corresponding to onset potential of 0.786, 0.879, 0.909, 0.998 and 1.008 V, respectively. The ORR activity of G-1100 is negligible. The ORR activity is increased when fluorine-doped carbon is produced. The Pt-like activity was found for FG-1100. The obvious improvement of the ORR activity is caused by the fluorine doping. Thus, two conclusions are found: one is the fluorine doping of carbon with improvement in ORR activity, the other is the high fluorine content with crumpled structure leading to Pt-like activity. Figure 6*b,c* provides a comparison of the electron transfer number along with the yield of HO_2_^−^ of all samples in 0.1 M KOH. The value of *n* for FG-1100 is 3.98–4.05 over the whole range of potentials, compared with the commercial Pt/C, indicating that the catalysed process of FG-1100 is the efficient four-electron pathway. It is noted that FG-1000, FG-1100 and FG-1200 show higher values of *n* than G-1100. This result suggests that the presence of the doped F further promotes the four-electron ORR process in alkaline media. The measured HO_2_^−^ yield for FG-1100 sample is lower than 4% over the whole range of potentials. From a practical point of view, this is very important, as the H_2_O_2_ generated in the two-electron process may degrade the catalyst layer and the proton exchange membrane. By contrast, the HO_2_^−^ yield for G-1100, FG-1000 and FG-1200 is higher, demonstrating its excellent electrocatalytic selectivity. This is consistent with the RDE results. The ORR parameters of different catalysts are summarized in electronic supplementary material, table S2.

The long-term durability is a major concern for evaluating the performance of ORR catalysts and much attention has been paid recently. The durability of the FG-1100 and Pt/C catalysts was estimated by using the accelerated durability test protocol by cycling the potential between 0.6 and 1.0 V. As shown in figure 6*d*, after 5000 cycles, the ORR half-wave potential of Pt/C underwent an obvious negative shift of 44 mV, whereas FG-1100 decreased only by 23 mV. Chronoamperometric testing was carried out to confirm the durability of FG-1100 and Pt/C with the potential holding at 0.6 V (versus RHE) in O_2_-saturated 0.1 M KOH. The chronoamperometric responses shown in figure 6*e* reveal that FG-1100 shows a 10% decrease in current density after 10 h, whereas commercial Pt/C decreases by 50% in current density. These results showcase the superior durability of FG-1100 compared to Pt/C with the potential for practical application.

For practical application in direct methanol fuel cells (DMFC), an ORR catalyst should possess outstanding tolerance and resistance to methanol crossover. Therefore, the chronoamperometric responses of FG-1100 and Pt/C were carried out by injecting methanol into the O_2_-saturated 0.1 M KOH electrolyte. As illustrated in figure 6*f*, both FG-1100 and Pt/C catalysts show a negative current when oxygen is bubbling, indicating ORR activity. After the injection of 3 M methanol, Pt/C showed an instantaneous change of current density from negative to positive, indicating methanol oxidation on the electrode. By contrast, the cathodic current of FG-1100 catalyst remained relatively stable after methanol injection, demonstrating a sign of excellent tolerance to methanol. These results once more showcase FG-1100 as an outstanding ORR catalyst with a much lower cost than commercial Pt/C catalyst in alkaline condition.

## 4. Conclusion

In summary, a highly efficient ORR FG catalyst has been prepared using a thermal pyrolysis approach and zinc fluoride (ZnF_2_) as F-doping precursor. When the pyrolysis temperature was 1100°C, the FG sample has higher fluorine amount and crumpled structure, which enables the catalyst to have more catalytic activity sites. The most active FG-1100 catalyst possesses prominent ORR catalytic activity with positive onset potential, high half-potential, high current density and excellent four-electron selectivity which is comparable with commercial Pt/C in alkaline electrolyte. FG-1100 catalyst also exhibited outstanding methanol-tolerant ability and durability superior to that of commercial Pt/C catalysts. The outstanding ORR catalytic activity of FG-1100 catalyst could be ascribed to a high density of F-doped active centres, which is derived from the higher fluorine amount and crumpled structure. The results suggested that FG catalyst can be developed as an efficient, cost-effective and durable catalyst for the commercialization of fuel cells and other electrochemical energy conversion devices.

## Supplementary Material

Fluorine-doped graphene with outstanding electrocatalytic performance for highly efficient oxygen reduction reaction in alkaline solution

## Data accessibility

The datasets supporting this article have been uploaded as the electronic supplementary material.
