# Supplementary material for: Fluorine-doped graphene with an outstanding electrocatalytic performance for efficient oxygen reduction reaction in alkaline solution
Source: R Soc Open Sci. 2018 Oct 3;5(10):180925. doi: 10.1098/rsos.180925 (PMC6227960; doi:10.1098/rsos.180925)
Supplement: Fluorine-doped graphene with outstanding electrocatalytic performance for highly efficient oxygen reduction reaction in alkaline solution [file rsos180925supp1.docx]

**Electronic Supplementary Material**

**Fluorine-doped graphene with outstanding electrocatalytic performance for efficient oxygen reduction reaction in alkaline solution**

Jiahao Guo,* Jianguo Zhang, Hanqing Zhao, Yongshuang Fang, Kun Ming, Hao Huang, Junming Chen, Xuchun Wang

*^a^* College of Chemistry and Materials Engineering, Anhui Science and Technology University, Fengyang, Anhui, 233100, P. R. China.

E-mail: guojiahao1974@163.com


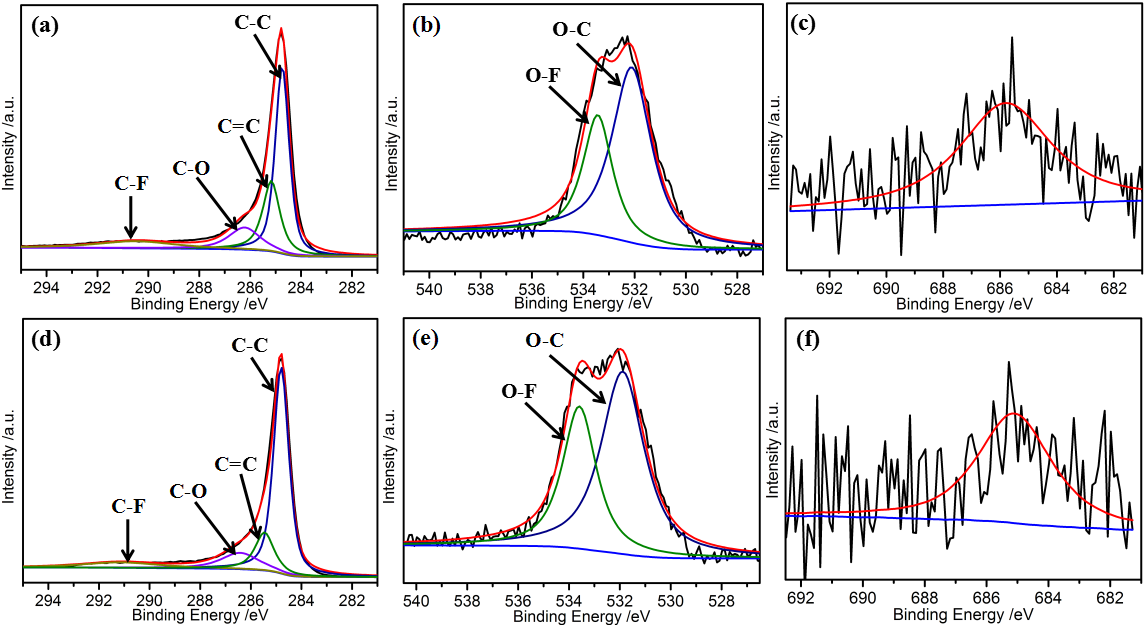


**Figure S1** (a) XPS -C1s, (b) XPS-O1s, (c) XPS-F1s spectra of FG-1000; (d) XPS-C1s, (b) XPS-O1s, (c) XPS-F1s spectra of FG-1200.


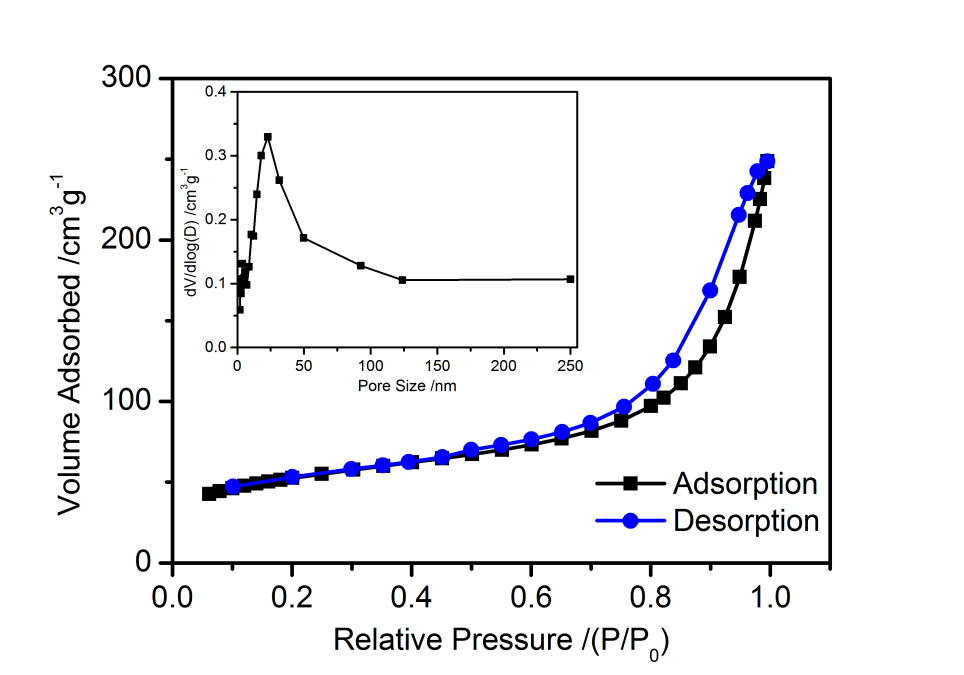


**Figure S2** Nitrogen adsorption-desorption isotherm of FG-1100, inset shows the BJH pore distribution


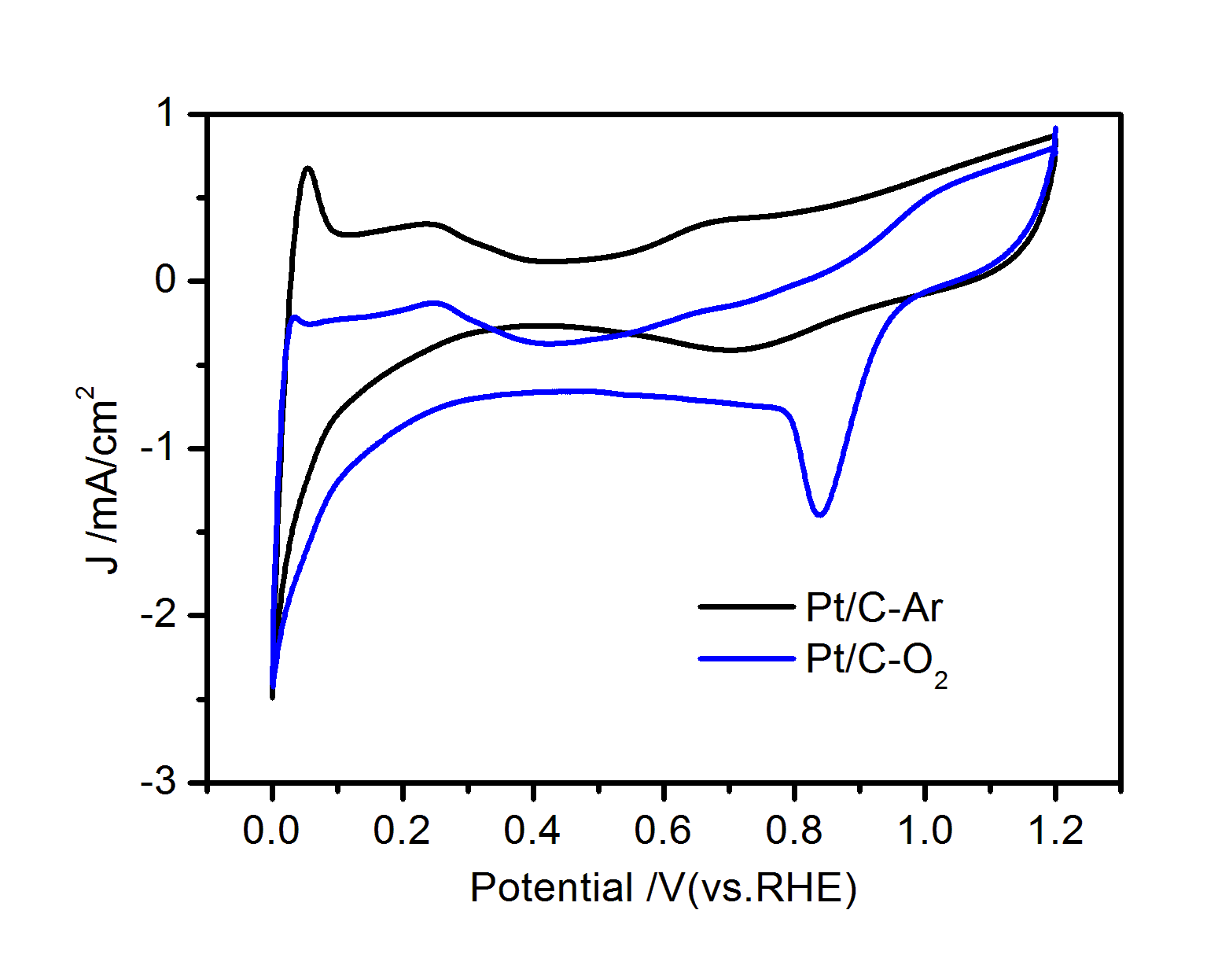


**Figure S3** CVs of commercial Pt/C in N_2_- and O_2_-saturated 0.1 M KOH.


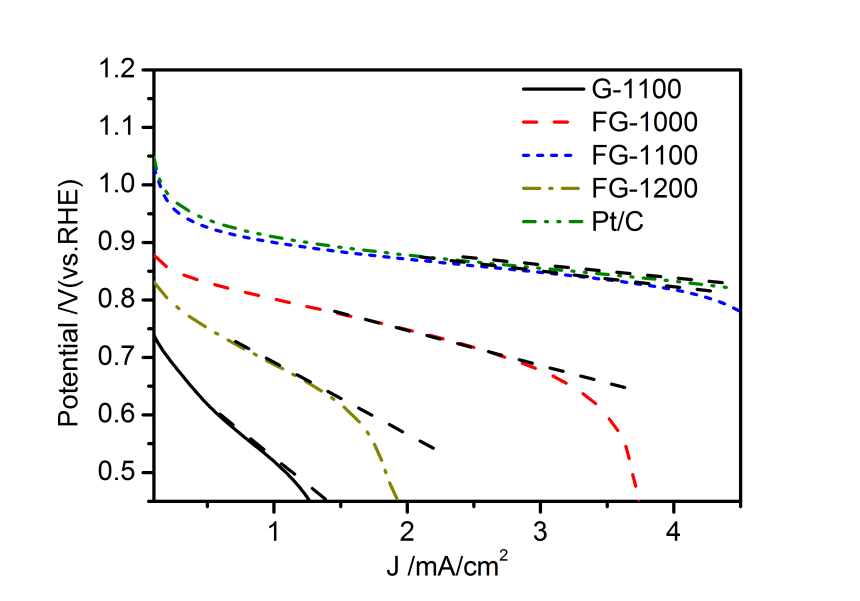


**Figure S4** The Tafel plots derived from Figure 5b in the low-current region.


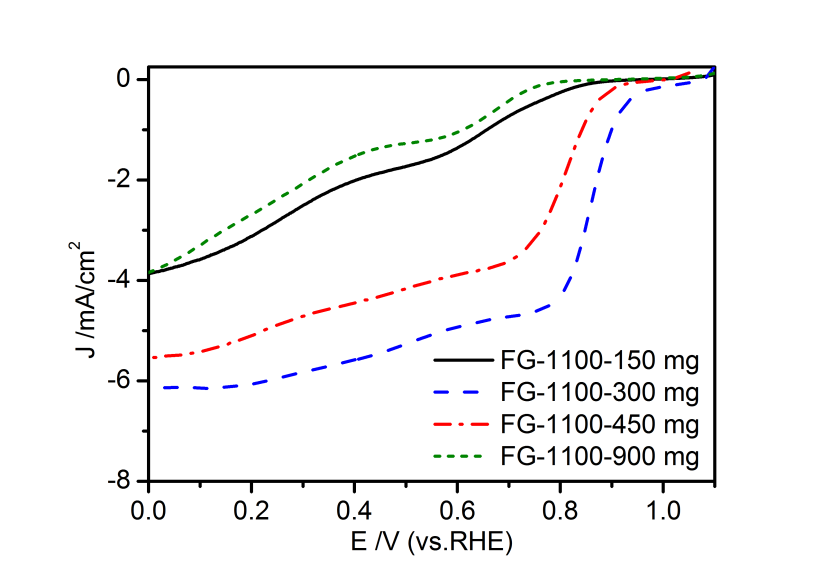


**Figure S5** LSV of FG-1100 with different amount of ZnF_2_ as F Source. For different FG-1100 samples, the amount of GO was 30 mg and the amounts of ZnF_2_ were 150 mg , 300 mg , 450 mg and 900 mg , respectively.


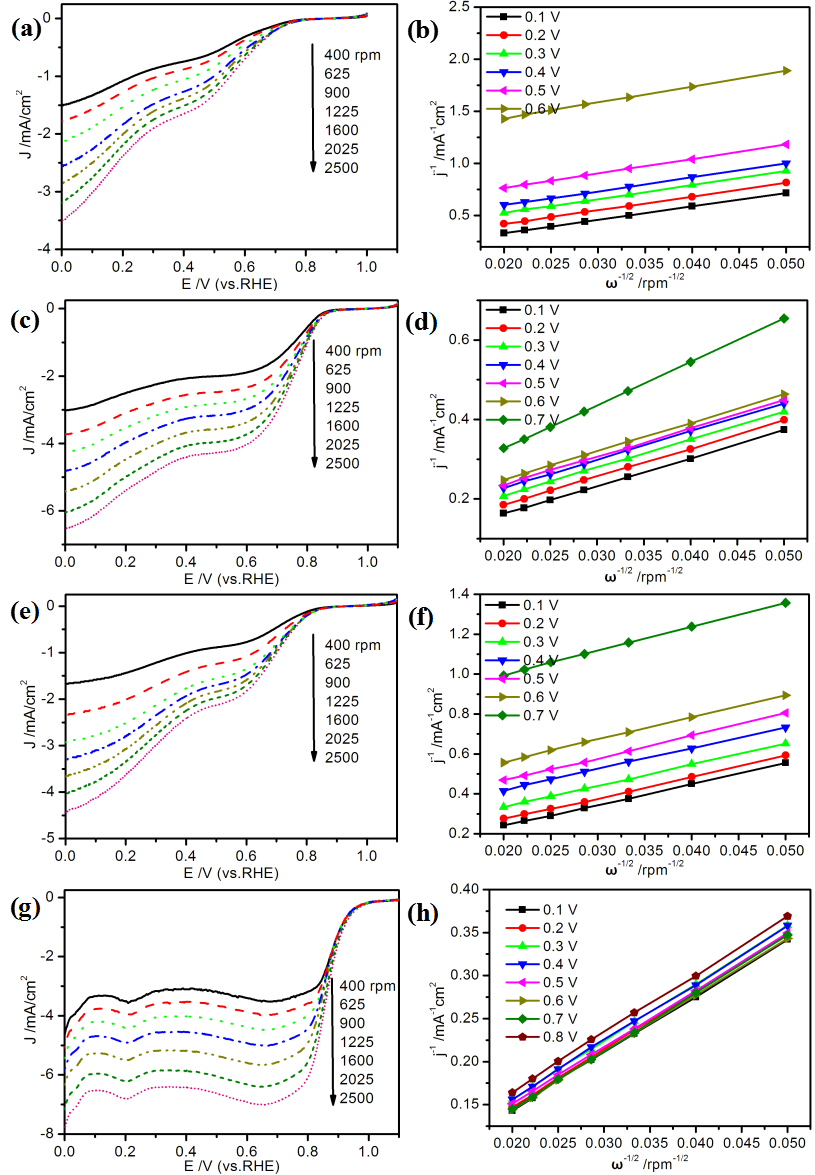


**Figure S6** (a) LSV of G-1100 at different RDE rotation rates. (b) Calculated K–L plots of ORR from G-1100. (c) LSV of FG-1000 at different RDE rotation rates. (d) Calculated K–L plots of ORR from FG-1000. (e) LSV of FG-1200 at different RDE rotation rates. (f) Calculated K–L plots of ORR from FG-1200. (g) LSV of commercial Pt/C at different RDE rotation rates. (h) Calculated K–L plots of ORR from commercial Pt/C.

**Table S1** Comparison of the electrocatalytic performance of FG-1100 and other metal-free

electrocatalysts toward ORR.

| Catalysts | Catalyst loading /mg cm^-2^ | Heteroatom content /at% | E_onset_ /V | E_onset_ (Pt/C)  /V in this article | E_1/2_ /V | E_1/2_ (Pt/C) /V in this article | J_L_/mAcm^-2^ | n |
| --- | --- | --- | --- | --- | --- | --- | --- | --- |
| P-doped  graphene [1] | 0.051 | 1.81 | 0.92 (vs. RHE) | 0.95 (vs. RHE) | Not mentioned | Not mentioned | Not mentioned | 3.0-3.8 |
| N-doped carbon nanosheets[2] | 0.2 | 48.7 | -0.076 (vs. Ag/AgCl) | -0.105 (vs. Ag/AgCl) | -0.210 (vs. Ag/AgCl) | -0.207 (vs. Ag/AgCl) | 5.79 | 3.9-4.0 |
| N-doped graphene foam[3] | 0.485 | 5.07 | 1.03 (vs. RHE) | 1.03 (vs. RHE) | 0.86 (vs. RHE) | 0.877 (vs. RHE) | 7 | 3.9 |
| B-doped reduced graphene oxide[4] | 0.1 | 2.9 | −0.05 (vs. Ag/AgCl) | −0.01 (vs. Ag/AgCl) | Not mentioned | Not mentioned | 4.97 | 4.16 at  −0.8 V |
| S-doped graphene nanoplatelets[5] | 0.075 | 4.94 | –0.22 (vs. Ag/AgCl) | –0.04 (vs. Ag/AgCl) | Not mentioned | Not mentioned | –4.47 at −0.8 V | 3.3 at  −0.6 V |
| B, N-doped Graphite[6] | 0.283 | N: 8.79 ± 1.17  B: 4.38 ± 0.87 | 0.047 (vs. Ag/AgCl) | 0.050 (vs. Ag/AgCl) | -0.147 (vs. Ag/AgCl) | -0.143 (vs. Ag/AgCl) | ~6 | 3.97 |
| F-doped graphene | 0.1 | 2.61 at% | 0.991 (vs.RHE) | 1.005 (vs.RHE) | 0.860 (vs.RHE) | 0.856 (vs.RHE) | 6.07 | 3.95-4.01 |

[1] Zhang C Z, Mahmood N, Yin H, Liu F, Hou Y L. 2013 Synthesis of Phosphorus-doped graphene and its multifunctional applications for oxygen reduction reaction and lithium ion batteries. *Adv. Mater.* **25**, 4932-4937.(doi:[10.1002/adma.201301870](https://doi.org/10.1002/adma.201301870))

[2] Yu H J, Shang L, Bian T, Shi R, Waterhouse G I N, Zhao Y F, Zhou C, Wu L Z, Tung C H, Zhang T R. 2016 Nitrogen-doped porous carbon nanosheets templated from g-C_3_N_4_ as metal-free electrocatalysts for efficient oxygen reduction reaction. *Adv. Mater.* **28**, 5080-5086. (doi:[10.1002/adma.201600398](https://doi.org/10.1002/adma.201600398))

[3] Zhou X J, Bai Z Y, Wu M J ,Qiao J L, Chen Z W. 2015 Dimensional porous N-doped graphene foam as a non-precious catalyst for the oxygen reduction reaction. *J. Mater. Chem. A* **3**, 3343-3350. (doi:10.1039/ C4TA06538G)

[4] Zhou Y Z, Yen C H, Fu S F, Yang G H, Zhu C Z, Du D, Wo P C, Cheng X N, Yang J, Wai C M, Lin Y H. 2015 One-pot synthesis of B-doped three-dimensional reduced graphene oxide via supercritical fluid for oxygen reduction reaction. *Green Chem.* **17**, 3552-3560.(doi:10.1039/ C5GC00617A)

[5] Jeon I Y, Zhang S, Zhang L P, Choi H J, Seo J M, Xia Z H, Dai L M. 2013 J. B. Baek, Edge-selectively sulfurized graphene nanoplatelets as efficient metal-free electrocatalysts for oxygen reduction reaction: The electron spin effect. *Adv. Mater.* **25**,6138-6145. (doi: 10.1002/adma 201302753)

[6] Jin J T, Pan F P, Jiang L H, Fu X G, Liang A M, Wei Z Y, Zhang J Y, Sun G Q. 2014 Catalyst-free synthesis of crumpled boron and nitrogen co-doped graphite layers with tunable bond structure for oxygen reduction reaction. *ACS Nano* **8**,3313-3321.(doi:10.1021/nn404927n)

**Table S2** The ORR parameters of different catalysts

| Catalysts | E_p_ of CV /V | J_p_ of CV /mA·cm^-2^ | E_onset_ /V | J_L_ at 0.2 V /mA·cm^-2^ | E_1/2_ /V | J_K_/mA·cm^-2^ | n |
| --- | --- | --- | --- | --- | --- | --- | --- |
| G-1100 | 0.514 | 0.25 | 0.792 | 2.01 | – | 3–8 | 1.76–2.04 |
| FG-1000 | 0.695 | 0.68 | 0.911 | 4.63 | 0.730 | 19–32 | 3.46–3.81 |
| FG-1100 | 0.725 | 1.67 | 0.991 | 6.07 | 0.860 | 33–52 | 3.95–4.01 |
| FG-1200 | 0.654 | 0.39 | 0.875 | 3.07 | – | 12–25 | 2.23–2.57 |
| Pt/C | 0.825 | 1.05 | 1.005 | 5.49 | 0.856 | 32–49 | 3.96–4.07 |
